# Intraoperative and Implant-Related Parameters of Isolated Intertrochanteric Rotational Osteotomy for Pathological Femoral Torsion: A Descriptive Single-Center Report

**DOI:** 10.7759/cureus.117494

**Published:** 2026-10-05

**Authors:** Ioannis Papadimitriou, Pavlos Sampatakakis, Maria Konsoula, Christian Lüring

**Affiliations:** 1 Department of Orthopedics, Klinikum Dortmund gGmbH - Klinikzentrum Mitte, Dortmund, DEU; 2 Department of Human Medicine, Faculty of Health, Witten/Herdecke University, Witten, DEU; 3 Department of Orthopedics, Christliches Klinikum Unna West, Unna, DEU; 4 Department of Plastic and Reconstructive Surgery, Helios Klinikum Krefeld, Krefeld, DEU

**Keywords:** femoral torsion, fixed-angle plate, implant configuration, intertrochanteric rotational osteotomy, joint-preserving hip surgery, operative time, perioperative parameters

## Abstract

Background

Isolated intertrochanteric rotational osteotomy (ITO) corrects pathological femoral torsion at the intertrochanteric level through a planar osteotomy stabilized with a fixed-angle plate. Functional results of joint-preserving femoral osteotomies appear regularly, yet the parameters that define how the operation is actually performed, including the rotational correction angle, the implant configuration, operative time, and length of hospital stay, are rarely quantified. This report documents these intraoperative and implant-related parameters in a procedurally homogeneous single-technique cohort.

Methods

Patients treated with isolated rotational or derotational ITO between 2002 and 2009 at one tertiary center were reviewed retrospectively. The descriptive variables fixed in advance were the intraoperative rotational correction angle, the implant configuration (plate angle, plate length, plate offset, and number of fixation screws), skin-to-skin operative time, and length of hospital stay. No inferential or outcome analysis was undertaken. Variables are summarized as mean ± standard deviation or as counts and percentages.

Results

Sixty patients were analyzed (42 women, 18 men; mean age 28 ± 13 years). A rotational osteotomy was carried out in 43 patients (71.7%), and a derotational osteotomy in 17 (28.3%); the operated side was left in 28 (46.7%) and right in 32 (53.3%). The mean rotational correction angle was 18 ± 9°. Fixation used a fixed-angle plate with a mean plate angle of 113 ± 11° and a mean length of 67 ± 10 mm, secured with a mean of 4 ± 1 screws. Plate offset was 10 mm in 35 patients (58.3%), absent in 16 (26.7%), 5 mm in five patients (8.3%), and 15 mm in four patients (6.7%). The mean skin-to-skin operative time was 124 ± 27 minutes, and the mean length of hospital stay was 12 ± 2 days.

Conclusions

This report quantifies the intraoperative and implant-related profile of isolated ITO in a single-technique cohort. The intraoperative correction angle averaged 18° (median 15°), an intraoperatively set correction rather than one referenced to a measured preoperative torsion, with wide individual variation; fixation followed a single fixed-angle principle using blade and angle plates of comparable geometry. Because preoperative torsion was not quantified radiographically in this cohort, the correction angle is reported as an intraoperatively set surgical parameter. These figures are descriptive and document how the procedure was executed at one center during this period; they are not outcome, survivorship, or safety measures.

## Introduction

Joint-preserving femoral osteotomy keeps the native hip and defers arthroplasty in younger patients, and the case for it usually rests on functional and survivorship data [1,2]. How the operation is performed has its own importance. The rotational correction that is set, the implant chosen to hold it, the time the procedure takes, and the days a patient spends in hospital shape surgical planning, theatre scheduling, implant selection, and training, yet these parameters are reported far less often than the scores that follow them.

Pathological femoral torsion is an extra-articular deformity that alters hip loading and has been associated with degenerative change in susceptible hips [3,4]. Intertrochanteric rotational osteotomy (ITO) addresses the deformity directly, outside the joint, through a planar osteotomy fixed with an angled plate [5,6]. Reports of the procedure concentrate on mid- to long-term function, often in mixed cohorts or alongside pelvic or intra-articular steps [7-9], and the intraoperative detail is usually compressed into a single methods sentence. The actual distribution of correction angles, the implant geometry used to secure them, and the perioperative metrics of the operation rarely reach the results.

This report sets out the intraoperative and implant-related parameters of a single-center cohort treated with isolated ITO for symptomatic femoral torsion. The variables of interest are the rotational correction angle, the plate configuration and fixation, operative time, and length of stay. The functional outcomes, the patient-reported scores, and the long-term safety profile of the same cohort are analyzed in a separate companion paper [10] and are deliberately left out here so that each report answers one question on its own terms.

This analysis draws on the same retrospective cohort as a separate, outcomes-focused manuscript that reports the functional and patient-reported results and the long-term safety profile in detail [10]. The two papers answer different questions. The companion paper covers function, patient-reported outcomes, regression, and complications; the present paper is confined to the intraoperative and implant-related parameters of the operation.

A separate e-poster addressing a methodological aspect of the same study cohort has been accepted for presentation at the Deutscher Kongress für Orthopädie und Unfallchirurgie (DKOU) 2026; the intraoperative and implant-related analysis reported here was not part of that e-poster.

## Materials and methods

Study design and ethical approval

This was a retrospective, single-center, descriptive cohort analysis of patients treated with isolated intertrochanteric rotational or derotational osteotomy between January 2002 and December 2009. The study followed the Declaration of Helsinki and was approved by the Ethics Committee of the Faculty of Medicine, University of Witten/Herdecke (approval no. S-120/2022) [11]; the committee waived written informed consent given the retrospective design. Reporting follows the Strengthening the Reporting of Observational Studies in Epidemiology (STROBE) statement [12].

Patient selection

Patients were eligible if they had undergone an isolated intertrochanteric rotational or derotational osteotomy for clinically symptomatic pathological femoral torsion and had operative records sufficient to document the index procedure. The indication rested on a comprehensive pre-operative clinical and functional examination: the hip rotational profile (internal and external rotation in flexion and extension), gait analysis including the foot progression angle, load-dependent symptoms, abductor function, the Trendelenburg sign, clinically significant limping, range of motion with side-to-side comparison, and leg alignment and weight-bearing. Surgery was indicated for symptomatic torsional malalignment on this assessment after exclusion of relevant alternative pathologies. Patients were excluded for prior hip surgery, any combined pelvic or femoral procedure, relevant coronal-plane deformity, neurological or systemic disease affecting gait, clinically significant acetabular pathology, or advanced osteoarthritis. These criteria produced a procedurally homogeneous cohort treated with one fixation principle.

Surgical technique

All operations used a lateral, proximal approach to the femur through an anterolateral interval that spared the surrounding muscle. A planar intertrochanteric osteotomy was cut perpendicular to the femoral shaft without wedge resection. Kirschner wires placed before the cut marked the intended rotation and let the correction angle be set under direct control. The proximal fragment was rotated to the planned position and fixed with a fixed-angle implant (blade plate or angled plate) and screws according to established principles of internal fixation​ [5,13,14]. The manufacturer and model of the implant could not be retrieved from the historical records. Mobilization began on the first postoperative day with free range of motion and no weight bearing; partial loading was withheld for about five weeks, after which weight bearing was advanced under physiotherapy guidance toward full loading after radiographic consolidation.

Recorded parameters

The parameters fixed in advance were the intraoperative rotational correction angle in degrees; the implant configuration, comprising the plate angle, the plate length, the plate offset, and the number of fixation screws; the skin-to-skin operative time in minutes; and the length of hospital stay in days. The operated side was recorded. Cross-sectional imaging for quantitative torsion measurement was not part of routine practice in this period, so the torsional indication rested on the clinical rotational profile and operative findings rather than on measured version.

Statistical analysis

The analysis was descriptive. Continuous variables are reported as mean ± standard deviation and, where the distribution was skewed, additionally as median with interquartile range and full range; categorical variables are reported as counts and percentages. No regression or time-to-event analysis was carried out. The associations between operative parameters and functional outcome, the patient-reported scores, and the complication and reintervention profile are addressed in the companion report and are not reproduced here.

## Results

Cohort

Sixty patients met the criteria (42 women (70.0%), 18 men (30.0%)). The mean age at surgery was 28 ± 13 years. A rotational osteotomy was performed in 43 patients (71.7%) and a derotational osteotomy in 17 (28.3%). The operated side was left in 28 patients (46.7%) and right in 32 (53.3%). The cohort characteristics are shown in Table 1.

**Table 1:** Cohort and Procedure Characteristics

Variable	Value
Number of patients	60
Female, n (%)	42 (70.0%)
Male, n (%)	18 (30.0%)
Mean age at surgery ± SD, years	28 ± 13
Rotational osteotomy, n (%)	43 (71.7%)
Derotational osteotomy, n (%)	17 (28.3%)
Operated side, left, n (%)	28 (46.7%)
Operated side, right, n (%)	32 (53.3%)

Rotational correction

The intraoperative rotational correction angle averaged 18° but varied substantially between patients (standard deviation 9°), reflecting the range of individual deformities. Because preoperative femoral torsion was not quantified by standardized cross-sectional imaging in this cohort (see Materials and Methods), this angle is reported as the intraoperatively set surgical correction and not as a correction relative to a measured baseline torsion.

Implant configuration and fixation

Fixation used a fixed-angle plate in every case. The mean plate angle was 113 ± 11°, and the mean plate length was 67 ± 10 mm. A mean of 4 ± 1 screws secured the construct. Plate offset varied: 10 mm in 35 patients (58.3%), no offset in 16 (26.7%), 5 mm in five (8.3%), and 15 mm in four (6.7%). The construct was therefore uniform across the cohort in plate type and dimensions, with variation confined to the plate offset and a narrow range of screw numbers. The implant parameters are summarized in Table 2.

**Table 2:** Intraoperative and Implant-Related Parameters Values are mean ± standard deviation or n (%). Parameters reflect documentation available in institutional operative records. All summary values in this table were previously reported descriptively in reference [10].

Parameter	Value
Rotational correction angle, degrees	18 ± 9
Plate angle, degrees	113 ± 11
Plate length, mm	67 ± 10
Number of fixation screws	4 ± 1
Plate offset, 10 mm	35 (58.3%)
Plate offset, none	16 (26.7%)
Plate offset, 5 mm	5 (8.3%)
Plate offset, 15 mm	4 (6.7%)
Skin-to-skin operative time, minutes	124 ± 27
Length of hospital stay, days	12 ± 2

Operative time and hospital stay

The mean skin-to-skin operative time was 124 ± 27 minutes. The mean length of hospital stay was 12 ± 2 days.

## Discussion

Principal findings

This cohort documents how isolated ITO was carried out rather than how patients fared afterward. Three features stand out. The rotational correction clustered near 18°, the fixation was uniform in principle, with a single fixed-angle plate type of consistent dimensions held by four screws on average, and the perioperative footprint was a procedure of about two hours followed by roughly 12 days in hospital. Taken together, these numbers describe a standardized operation rather than a heterogeneous collection of techniques.

Intraoperative parameters in context

The correction averaged 18° with wide variation between patients. Because preoperative torsion was not quantified by standardized imaging in this cohort, this angle reflects the surgeon’s intraoperative setting rather than a correction measured against a defined baseline, and the spread is consistent with a range of individual deformities rather than with variation in technique. The fixed-angle construct, with a plate angle near 113° and a length close to 67 mm, matches the blade- and angle-plate implants in use for intertrochanteric femoral osteotomy during the study period [5,13]. The offset distribution, dominated by a 10 mm offset or none, indicates that medial displacement was applied selectively rather than as a routine step.

The value of reporting these parameters is practical. A surgeon planning an intertrochanteric rotational osteotomy needs to know the correction angles that arise in practice, the implant geometry that secures them, and the theatre time and inpatient stay the procedure demands. A functional score supplies none of this, and the present report brings these figures together for one homogeneous series.

Comparison with the literature

Direct comparison with the literature is constrained by differences in anatomical level and indication. Many reported torsional osteotomies are performed at the distal femur or for patellofemoral disorders [5], whereas the present series is exclusively intertrochanteric and performed for hip-level torsional symptoms. The closest comparators are proximal femoral rotational osteotomies: Buly and colleagues and Rigling and colleagues describe femoral derotation at the proximal and subtrochanteric level for version abnormalities [7,8], and the transtrochanteric rotational osteotomy described by Sugioka shares the proximal location, though it was developed for a different indication [13]. Across these proximal series, correction magnitudes and fixation principles are broadly comparable to those reported here, yet operative time, implant configuration, and length of stay are seldom quantified, which is the gap this descriptive report addresses. Long-term follow-up of any torsional osteotomy, at any level, remains uncommon [15].

Perioperative metrics

A skin-to-skin time near two hours is consistent with an open intertrochanteric osteotomy that includes exposure, controlled rotation, and plate fixation. The mean inpatient stay of 12 days reflects the perioperative and rehabilitation practice of a 2002 to 2009 European setting and should not be read against contemporary fast-track pathways, under which length of stay after comparable osteotomy is considerably shorter. The figure is reported as a historical descriptor of this cohort, not as a target for current care.

Strengths and limitations

The strength of this report is the homogeneity of the series: one center, one fixation principle, and a single osteotomy concept across all 60 patients, which makes the parameter distributions interpretable. The limitations are clear. The design is retrospective, and the data are historical, so the perioperative figures reflect the practice of their period. Operative time and length of stay were recorded as routine clinical variables and were not stratified by correction direction, and blood loss, transfusion, and intraoperative complication data were not available for analysis. The single-center setting limits external validity, and the fixed-angle plate documented here has since been joined by other fixation options. These figures describe one series and are not presented as standards. In particular, preoperative femoral torsion was not quantified by standardized cross-sectional imaging, so the reported correction angle is an intraoperative surgical setting rather than a value referenced to a measured baseline, and its wide spread should be read in that light.

## Conclusions

This report contributes a documented, internally consistent record of how isolated intertrochanteric rotational osteotomy was performed in one homogeneous single-technique cohort: a single fixed-angle plate construct of consistent dimensions, an intraoperative correction that averaged 18° with wide individual variation, and a perioperative course tied to the practice of its period. Its value is as a transparent procedural reference for a technique whose operative detail is rarely quantified; its limits are equally clear, since the data are retrospective and historical, the preoperative torsion was not measured, and the parameters were neither stratified nor linked to outcome. Prospective, imaging-anchored data would be needed to turn this descriptive record into evidence on which correction magnitudes and fixation choices could be based.
